# Interactive responses of root and shoot of camphor tree (*Cinnamomum camphora* L.) to asymmetric disturbance treatments

**DOI:** 10.3389/fpls.2022.993319

**Published:** 2022-11-29

**Authors:** Hongbing Wang, Yonghong Hu, Jun Qin, Chenbing Guo, Duorun Wu, Qiang Xing, Lianlian Pan, Kangsheng Xia, Yajun Shen, Jingjing Guo, Ran Jiang

**Affiliations:** ^1^ College of Life Sciences, Shanghai Normal University, Shanghai, China; ^2^ Shanghai Engineering Research Center of Plant Germplasm Resources, Shanghai, China; ^3^ Shanghai Chenshan Botanical Garden, Shanghai, China

**Keywords:** root-shoot balance, camphor tree, coefficient of asymmetry, minirhizotron, non-structural carbohydrates (NSC), root design, urban forest

## Abstract

Plant root and shoot growth are closely interrelated, though the connotation of root–shoot balance should not be limited to their connectivity in biomass and physiological indicators. Their directional distribution of mass in architecture and the resulting root–shoot interactions are the keys to understanding the dynamic balance of the below- and above-ground organs related to tree anchorage. This study focuses on the 4-year-old camphor tree (*Cinnamomum camphora* L.) as a system to observe the biomass distribution in response to the asymmetric disturbance treatments of biased root (BRT), inclined trunk (ITT), and half-crown (HCT) in a controlled cultivation experiment using the minirhizotron technique. We found an inverse relationship of biomass distribution of crowns to roots in BRT and opposite asymmetries of roots with crowns in response to the ITT and HCT treatments. We also observed higher net photosynthesis rate (*P_n_*), water use efficiency, and chlorophyll content in the leaves on the side opposite the lean in ITT, and higher *P_n_*, transpiration rate, and chlorophyll content on the root-bias side in BRT, which is consistent with the nutrient allocation strategies of allocating nutrients across plant organs in an optimal way to obtain ‘functional equilibrium’ and adapt to the stressed environment. Furthermore, the asymmetrical growth transformation of first-level branch length from the root-bias side to the opposite side in BRT, and a similar transformation of root length from the crown-bias side to the opposite side in HCT, imbues further theoretical support of the nutrient allocation strategy and the biomechanical stability principle, respectively. In summary, this study is the first to identify opposite interaction between below- and above-ground biomass distributions of the camphor tree. The findings enrich the connotation of root–shoot interactions and help to realize root design for the silviculture management of urban forests.

## 1 Introduction

Plant root and shoot growth are closely interrelated (Horst and Hoffmann, 1967) and follow a dynamic balance and optimal process that change with age, the nutrient and water availability in soil, and the light intensity on the canopy (Iwasa and Roughgarden, 1984; Velten and Richter, 1995). The strongest correlations are found between fine root surface area and leaf area, as well as below- and above-ground biomass (O’Grady et al., 2006). For example, there are significant correlations between biomass and surface area of fine roots as well as those of leaves in *Larix gmelinii*, a tree native to China (Meng et al., 2018). A similar strong positive correlation is found between crown pruning and the rejuvenation of shoots and rooting (Wilson, 1999); specifically, root loss from root pruning can slow crown growth (Geisler and Feree, 1984; Koeser and Stewart, 2009), as reported in the tree *Cunninghamia lanceolata* (Dong et al., 2019).

The below- and above-ground relationship is explained from the perspective of biomass and physiology, and root/shoot biomass ratio (R/S) is often used to express their physical balance in biomass (Wilson, 1988; Watson, 1991; Poorter et al., 2012; Pryor and Watson, 2016; Askari et al., 2017). The R/S is influenced by internal and external factors and varies among species, owing to the allometric growth pattern (Horst and Hoffmann, 1967; Askari et al., 2017). For example, shade-tolerant species often have higher R/S than light-demanding species (Cao and Ohkubo, 1998). Tropical–subtropical moist forests or plantations have the lowest R/S (0.205) among global forest vegetation types (Mokany et al., 2006). In China, R/S is lower in conifer forests than in broadleaved forests, in evergreen forests than in deciduous forests, and in plantations than in natural forests (Luo et al., 2012; Meng et al., 2018). R/S also decreases with increasing tree height and diameter at breast height (Cao and Ohkubo, 1998; Marziliano et al., 2015; Ledo et al., 2018), and flexed plants have higher R/S than erect plants (Gartner, 1994). Further, R/S is negatively related to mean annual precipitation and temperature (Mokany et al., 2006), as well as to soil-root plate depth (Nicoll and Ray, 1996). Given the ability to adapt to ecological environments, R/S significantly increases under drought stress (Ledo et al., 2018), which can be explained by nutrient allocation strategies. Nutritional resources (e.g., carbon and other photosynthetic products) are allocated more to the roots when mineral elements in the substrate are scarce, which is a strategy to increase the R/S ratio and optimize root morphology (Hermans et al., 2006).

The connotation of root–shoot balance should not be limited to the mere root to shoot biomass partitioning, since the latter is a poor indicator of tree stability (Nicoll et al., 1995). Reduced/restricted root systems, asymmetric root systems, and asymmetric crowns can reduce stability (Watson et al., 2014; Tomao et al., 2015), indicating the importance of architectural elements. For example, wind-induced asymmetries can affect the below- and above-ground organs of trees, and a tree with an asymmetrical or restricted root system may be less stable. From a biomechanical perspective, tree growth directly correlates to the root–shoot mechanical balance. Morphological responses of the root system may occur when a tree is subjected to certain mechanical stresses (Tamasi et al., 2005). In this process, trees can adjust the internal structures using an adaptive growth strategy of thigmomorphogenesis in response to external mechanical stimuli such as wind stress (Jaffe, 1973; Kontogianni et al., 2011). The resulting morphologies of leaves, stems, and root systems change in relation to size and distribution, showing asymmetries on the windward and leeward sides of the tree (Gardiner, 1995; Salekl et al., 2017). Notably, the wood on the leeward side is denser and thicker as to provide greater compression support (Coutts, 1986; Nicoll et al., 1995); and the root system on the windward side is longer and more extensive with larger stumps to provide a greater tensile strength for anchorage (Young and Perkocha, 1994; Danjon et al., 2005; Peltola, 2006; Yang et al., 2017; Stubbs et al., 2019).

Tree asymmetry is common around the world. Crown asymmetry is always found in natural forests because of crown avoidance to limit competition with neighbors (Getzin and Wiegand, 2007). In cities, tree crowns are often biased away from buildings and root systems develop away from gray infrastructures (Kontogianni et al., 2011; Bobrowski et al., 2017) to maximize growth potential. Generally, different viewpoints on the root–shoot architectural relationships exist. Some studies have found that root spread responds to mechanical stimuli transmitted from the shoots (Stubbs et al., 2019), and the whole root system responds spatially to an asymmetric crown by shifting root biomass to the opposite side of the tree for balance (Hardiman et al., 2017; Kolb et al., 2017). Other studies suggest that one-sided crown development is associated with one-sided formation of the root system (Horst and Hoffmann, 1967). For example, a positive correlation between the root biomass direction and crown orientation of *Tilia cordata* is found in the Morton Arboretum, USA (von der Heide-Spravka and Watson, 1990). Additionally, *Picea sitchensis* trees exhibit intraspecific variation in the direction of root biomass allocation, with biomass being distributed toward the leeward (Nicoll et al., 1995; Nicoll and Ray, 1996) or the windward side (Coutts, 1986; Stokes et al., 1995); and toward the downslope (Nicoll et al., 1995) or upslope side (Nicoll and Ray, 1996; Danjon et al., 2005; Di Iorio et al., 2005; Nicoll et al., 2006) based on environmental factors. The interactions between tree root and shoot in response to mechanical perturbations are influenced by a number of biotic and abiotic factors that are extremely complex and difficult to analyze. Therefore, we should clarify the biological internal relations of root–shoot architecture prior to eliminating the interference of external factors. The contentious results regarding aboveground growth responses to the root asymmetry indicate a need for further exploration. We should examine the morphological correlations between below- and above-ground organ interactions of different tree species under diverse asymmetric treatments.

Compared to the crown, the tree root system architecture is relatively difficult to access and evaluate on account of its position in the soil (Barthélémy and Caraglio, 2007). The minirhizotron method is well suited for studying fine root dynamics because it includes direct *in situ* and non-destructive visualization, and dynamic spatial and temporal monitoring of root growth (Krasowski et al., 2010; Gray et al., 2012; Kou et al., 2018; Ohashi et al., 2019). This study used the minirhizotron technique to monitor the dynamics of spatial and temporal growth of fine roots.

The camphor tree (*Cinnamomum camphora* L.), native to China and Japan, is an important ornamental species in subtropical evergreen broad-leaved forests and is widely cultivated in East and South Asia (Shi et al., 2009), especially in the southern region of the Yangtze River, China. Camphor is the predominant urban street tree in Shanghai, comprising 40% of the total 0.92 million trees (Shen, 2012). The camphor tree has an extensive shallow root system concentrated in the shallow soil layer that extends over larger horizontal distances. In this study, we used the camphor tree as a test species to identify the biomass distribution in response to the asymmetric distribution treatments in roots, trunks, and crowns under controlled conditions. Knowledge of the directional relations of root and shoot biomass would enrich the connotation of root–shoot balance and help to realize potential root design in silviculture management of urban forests.

## 2 Material and methods

### 2.1 Study site and test species

The study was conducted at the Fengxian Campus, Shanghai Normal University (N 30°50′32.26″, E 121°30′38.96″) in the south end of Shanghai, China. The area is characterized by low-lying alluvial plains (3–5 m a.s.l.) and a northern subtropical humid monsoon climate. The average annual temperature is 17.8°C, average annual precipitation is 1660.8 mm, and dominant wind direction is from the southeast during the growing seasons (Shanghai Municipal Bureau of Statistics, 2021).

### 2.2 Experimental design

**Figure 1:**
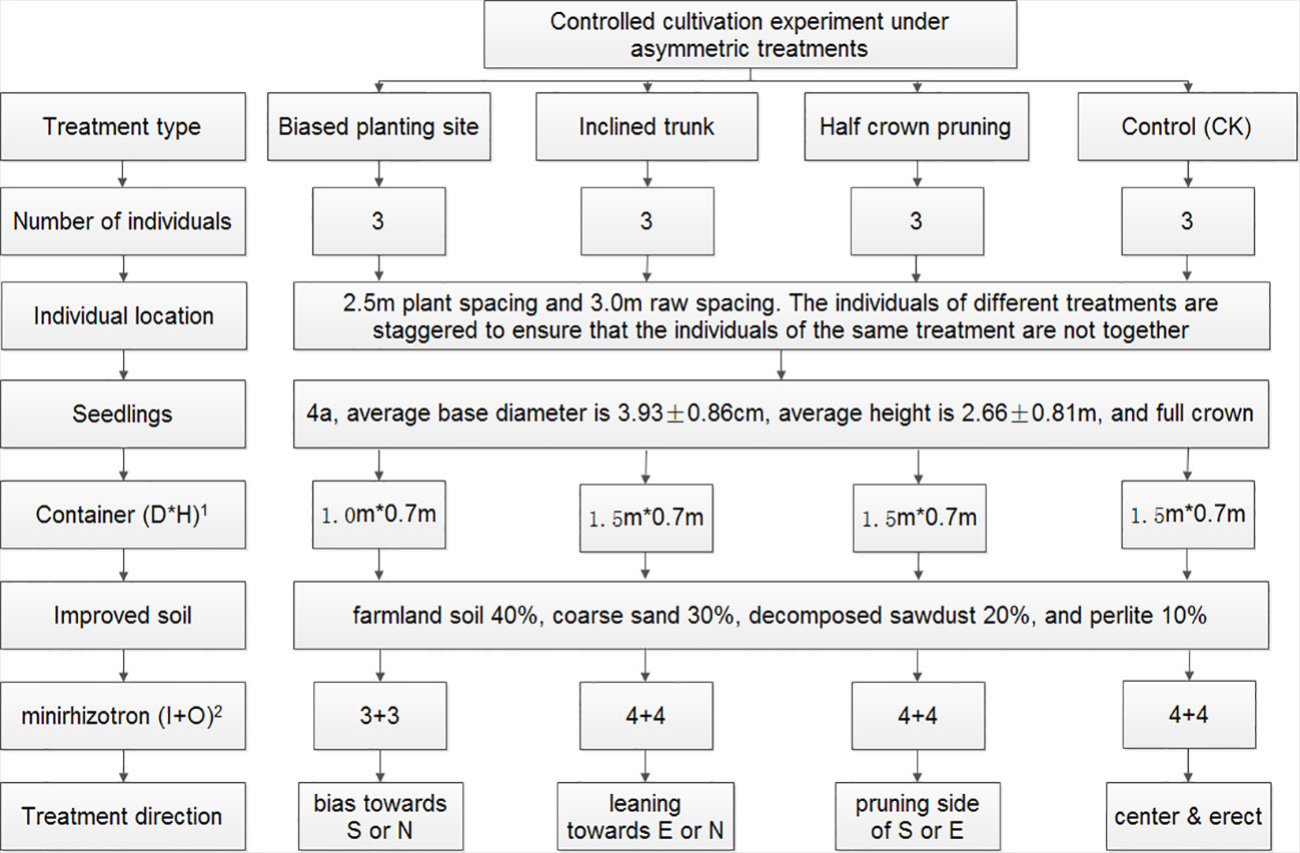
Experimental design of the controlled cultivation. ^1^D is the diameter of container; H is the height of container; ^2^I and O are the locations of the inner circle and outer circle of the minirhizotron tubes, respectively.

In cities, street trees often face limited growing space owing to impervious surfaces such as concrete, and one-sided constrained spaces such as those adjacent to the road, in the narrow road isolation belt, by the waterfront, or adjacent to buildings. Based on the potentially asymmetric growth situations, we designed the following three root-stem-crown, respectively, asymmetry treatments: biased-root treatment (BRT), planting site was adjacent to one side of container to establish asymmetric rooting space; inclined-trunk treatment (ITT), tree trunk was manually bent toward one side; and half-crown treatment (HCT), one half of the crown was pruned of the lateral branches to reserve the other half of the crown. In addition, trees with no asymmetric treatment were treated as the controls (CK). Photos of the treatments applied are provided in **Appendix 1**; a detailed experimental design is outlined in **Figure 1**.

**Figure 2:**
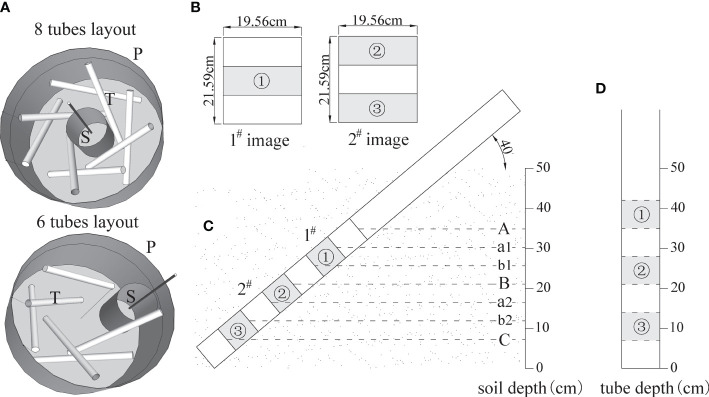
Minirhizotron tube layout, image subsample belt design, and corresponding soil layers. **(A)** rhizotron tubes layout, in which 8 tubes were arranged for ITT, HCT, CK, and 6 tubes for BRT; P ~ container, T ~ rhizotron tube, S ~ planting site; **(B)** distribution of subsample belts; **(C)** image location; **(D)** depth of subsample belts in tube; (A, B) are the top and bottom lines of the 1^#^ image; (B, C) are the top and bottom lines of the 2^#^ image; a1 and b1 are the three-equal-division lines in 1^#^ image; a2 and b2 are the three-equal-division lines in 2^#^ image; ①-③ are the three subsample belts from top to bottom in one tube.

The experiment was implemented in April 2019. All the used 4-year-old saplings were healthy with full crowns, straight stems, and intact soil balls. The containers, made of reeled porous PVC and shaped into cylinders, had a diameter of 100–150 cm and height of 70 cm (**Figure 1**) and were filled with soil up to ~60 cm in depth. The rhizotrons were installed at the time of sapling planting. The layout of rhizotron was designed as two tubes in one direction, one inside and one outside, and together eight tubes were arranged in four directions, showing evenly intertwined distribution around the central planting site (**Figure 2A**). Eight tubes were layout for one tree in ITT, HCT, and CK. The exception was that, for the tree planted close to one side (BRT), six tubes were necessary to be layout in other three directions. Three individuals per treatment including control were selected, for which a total of 90 rhizotron tubes were installed. In addition, one sapling, grown from spare saplings, was supplemented with 8 tubes for ITT in January 2020 to substitute one sample tree that exhibited weak growth in 2019. The tubes were inclined 45° clockwise and wrapped heavily with black tape above the soil surface to exclude light, which open ends were filled with a rubber stopper and covered with self-sealing aluminum bags to exclude light and water. Three months after planting (July 2019), the trunks were inclined up to 65° dip angle for ITT and half of lateral branches were removed for HCT. Six months after planting (October 2019), soils settled down to ~50 cm depth, and we simultaneously measured the dip angle of each tube, which was eventually stabilized at ~40°.

To counteract the influences of environmental factors such as solar radiation, wind loading, and soil heterogeneity, we employed a controlled cultivation experimental design, which included the following 10 measures: a) the saplings had similar base diameter and tree height; b) containers were of the same size to ensure similar rooting space; c) container bottoms included root-blocking plates and the ground was an impermeable concrete or brick surface to avoid underground root penetration; d) soil conditions, composition and depth, were the same; e) each sapling was at a similar distance from surrounding buildings and other obstacles; f) the individual layout exhibited alternate distribution to avoid any disturbances due to any uneven environmental conditions; g) the individuals showed diverse directions of asymmetric treatment within one treatment type; h) uniform management processes such as irrigation, fertilization, and pest control were employed; i) timely weeding and mulching with three-centimeter-thick bark layer to prevent interference from other plant roots; and j) same data collection schedules of below- and above-ground organs were followed.

### 2.3 Data collection of fine roots and crown morphology

The data collection, namely rhizotron image scanning, root tracing, and crown measurement, commenced from November 2019 to March 2022. Data were collected at five times in one year, that is, in March, May, July, September, and November. We failed to collect some data on the roots and shoots in March 2020 and on the branches in September 2020, March 2021, and March 2022 because of the serious coronavirus pandemic that began in March 2020. Altogether eleven datasets were collected for the traits of root systems and eight datasets collected for the traits of branches.

#### 2.3.1 Rhizotron image data on fine roots

In this study, fine root growth dynamics were monitored using the CI-600 *In-Situ* Root Imager (CID Inc., Camas, WA, USA). A reference point was drawn on the upper middle part of each tube end to mark a permanent start-scanning position. The instrument was calibrated before scanning. The Minirhizotron ~360° rotating scanner was placed at a vertical depth of approximately 50 cm, and two high-resolution digital images (19.56 cm × 21.59 cm, 100 dpi) were captured at the upper tube depth of 28.59–50.18 cm and lower tube depth of 7–28.59 cm, named 1^#^ image and 2^#^ image respectively (**Figures 2B, C**). A total of 196 images were collected each time, resulting in 2140 images taken altogether during the observation period. Three subsample belts were extracted from the two images within each tube (**Figures 2B–D**). Two horizontal reference lines were added and segmented into three equal belts in each image. The middle belt of the upper 1^#^ image was considered as the upper subsample belt (① in **Figure 2**). The upper and lower belts of the lower 2^#^ image were sampled as the middle and lower subsample belts respectively (② and ③ in **Figure 2**). Each subsample belt was 19.56 cm × 7.20 cm and 140.83 cm^2^ in surface area. All subsample belts were cut out from the same locations in all images. The 3210 subsamples had a collective image area of 452,070.7 cm^2^, which accounted for 50% of the total image area.

In the laboratory, the length and diameter of every visible root segment in each subsample belt were manually traced and analyzed using the WinRHIZOTron MF 2018a software (Regent Instrument Inc., Québec, Canada). The root length (RL) and surface area (RSA) were automatically calculated by the software.

Data quality of the manual root tracing has a direct impact on the experimental conclusions; thus, calibration was necessary prior to data analysis. The data fluctuation between two time points was due to the following: (1) image quality, which directly influences tracing results, was affected by the soil moisture and soil stability around the tubes, and therefore, root scanning was conducted within at least three days after rain; and (2) professional operation strongly enhances tracing accuracy. Although small errors are unavoidable, improved data processing and calibration were employed to limit errors within a controllable range. To minimize variation due to human error in root tracing, the effective countermeasures included:

1) Tracer training. All the technicians were trained to compare root tracing results using the same representative images with numerous and colorful roots before formal root tracing, and to analyze questionable image sections along unified standards. Two experienced tracers were responsible for completing the work to reduce the errors caused by more participants when the workload was small, and they played the main roles, while the other participants played supplementary roles, when the workload was large. Images from the same sapling were assigned to the same participant. Random cross-check was employed during the middle and late stages of image analysis.

2) Determining the standards. Only the live roots were traced, excluding dead roots and other impurities. The tracers were trained to identify live and dead roots by color and accurately interpret the root shape and size. The uncertain roots were re-judged based on comparison with the previous data set (i.e., previous images) to ensure the right position, right mark, and right growth pattern. Root length and thickness were adjusted according to a specific root shape to maintain a consistent and stable tracing hardness and margin.

3) Auxiliary calibration. After tracing each subsample belt, the values of root traits were compared with the previous corresponding data. The values of fine roots are expected to increase, to keep constant, or to decrease. A small decline was possible during the non-growing season or under unfavorable environments; however, a reasonable explanation for the abnormal values was required. Correction was needed in cases with human error.

#### 2.3.2 Morphological data of the crown

All the first-level branches were measured right after planting. During the growth process, the new healthy and stout first-level branches were additionally sampled and measured to ensure continuous tracking. The second-level branches were sampled in four directions, with ≥3 repetitions. If the tracked branch was damaged due to death or braking off, additional sampling was conducted immediately from new second-level branches to maintain a consistent number of repetitions. The third-level, fourth-level, and fifth-level branches were sampled in a similar process.

Measured indicators included tree height, base diameter, diameter at breast height, crown width (four directions), base diameter, and length of sampled branches.

### 2.4 Physiological data collection

#### 2.4.1 Photosynthetic parameters

A portable LI-6400 photosynthesis system (Li-Cor, Lincoln, NE, USA), equipped with a 6400-02B LED red/blue light house to control light intensity, was used to measure the photosynthetic characteristics of the trees (Huang et al., 2010). The leaves on the biased and opposite sides of all treatments were sampled and measured between 9:00 and 11:00 in August 2021. The net photosynthesis rate (*P_n_*), and transpiration rate (*T_r_*) of leaves were recorded. Water use efficiency (WUE) was calculated as *P_n_*/*T_r_* (Mielke et al., 2005).

#### 2.4.2 Leaf chlorophyll content

A chlorophyll meter (SPAD-502Plus; Konica-Minolta, Japan) was used to measure the chlorophyll concentration (Lichtenthaler and Wellburn, 1982 in ten leaves randomly sampled from each the biased and opposite sides of each sapling in mid-August 2021.

#### 2.4.3 Leaf NSC content

Thirty leaves were randomly sampled from each the biased and opposite sides of each sapling in late-August 2021. Leaf pre-treatment was performed according to Li et al. (2016). The standard anthrone colorimetric method was used to measure the content of soluble sugar and starch (Li et al., 2016; Dong et al., 2019). The content of non-structural carbohydrates (NSC) was estimated as the sum of the contents of soluble sugars and starch.

#### 2.4.4 Leaf nitrogen concentration

Leaves were randomly sampled from each the biased and opposite sides of each sapling in October 2021. Sample extraction was performed using sulfuric acid following the protocol similar to that used for soil samples (Abrams et al., 2014; Huang et al., 2018). The K9840 Kjeldahl Nitrogen Analyzer (Hanon Instrument Co. LTD, Jinan, China) was used to measure the total N concentration using the Kjeldahl method.

#### 2.4.5 Leaf SOD activity

In early October 2021, leaves were randomly sampled from each the biased and opposite sides of each treatment, and prepared by homogenizing 0.1g of frozen leaves in 1mL of extraction buffer (phosphate buffer saline, PBS). Homogenate was centrifuged at 8,000 rpm and 4°C for 20 minutes and supernatants were collected as enzyme extract. Three ml enzyme reaction mixture was reacted to 0.1 ml enzyme extract. The mix was vortexed and kept for 20 minutes. SOD activity was analyzed using a SOD assay kit (Chundubio, Wuhan, China) and then measured at 560 nm absorbance as ability to inhibit photochemical reduction of nitro-blue tetrazolium (NBT).

### 2.5 Data analysis

**Figure 3:**
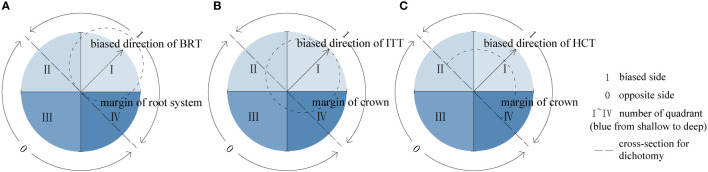
Schematic diagrams of asymmetric treatments. **(A)** The BRT treatment, **(B)** the ITT treatment, **(C)** the HCT treatment.

To analyze the asymmetries of roots and shoots, the directions of asymmetric treatments were defined as outlined in **Figure 3**: *1* represents the biased side with the larger mass of treated organ, that is, the side opposite to the side that stem base close to for BRT, the trunk leaning side for ITT, and the reserved canopy side for HCT; *0* represents the side opposite to *1*. Dichotomy analysis involves *1* and *0*, which represent each cross-section as two equal parts. The directions of other organs follow the above definitions in each treatment type. All the directional analysis of root–shoot architecture and physiological indexes conformed to the above definitions.

Crown asymmetry index (*CAI*) was first proposed by (Curtin (1970). (Kong et al. (2021) summarized and adapted the *CAI*s, of which the *CAI*_13_ was adapted from the simple bilateral symmetry measure.


(1) $$CAI_{13}\;=\;\frac{1}{N_{p}}\sum_{i=1}^{N_{p}}\frac{\left|\overset{\leftarrow }{R_{i}}\;-\;\vec{R_{i}}\right|}{\overset{\leftarrow }{R_{i}}\;+\;\vec{R_{i}}}$$

where *N_p_* is the number of paired crown radius measurements,

$\overset{\leftarrow }{R_{i}}$ and $\vec{R_{i}}$ represent the *i* ^th^ measurement of paired radii on two opposite sides of the crown.

In similar, we aimed to find the asymmetry of tree in one pair of directions. The asymmetry can be explained by crown traits and root traits. To quantify the bidirectional asymmetry of tree, a coefficient of asymmetry (CoA) was defined as the ratio of the difference between the two-sided variables to their mean. For example, the CoA of RSA was defined as,


(2) $$CoA\;=\;(S_{1}\;-\;S_{2})/\bar{S}$$

where *S_1_* and *S_2_* represent the RSA of the *1* and *0* side, respectively. $\bar{S}$ is the average of *S_1_* and *S_2_*. The root system is symmetric in RSA when CoA = 0, or asymmetric if CoA ≠ 0. The asymmetric direction is toward the *1* side when CoA > 0, and opposite to the *1* side when CoA< 0. Similar conventions were used to define the CoA of root and branch lengths.

All statistical analyses were performed in the SPSS 25.0 statistical software (IBM Corp., Armonk, NY, USA). Before choosing statistical criteria, all data were checked for the normality distribution and Homogeneity of Variances. For the normal distributed and homoscedastic data, T test and one-way ANOVA test were used for two groups (bidirectional 1^st^ branch traits, CoA in root surface area between groups) and more groups (total nitrogen and soluble sugar among treatments) respectively, and Welch test was adopted for the normal distributed and heteroscedastic data (bidirectional 2^nd^ and 3^rd^ branch traits in ITT). For the non-normal data, exponential transformation was conducted for the CoA of RL in HCT before analysis. For other non-normal data, nonparametric statistical methods were applied including Mann–Whitney test and Kruskal–Wallis test for two group (bidirectional root traits) and more groups (root and branch traits among the treatments) respectively. Statistical significance was defined as *p<* 0.05. The analysis of root and branch traits was performed using two-tailed Pearman’s correlation test.

## 3 Results

### 3.1 Effects of asymmetric treatments on tree growth

Among the asymmetric treatments, the lowest RL was found in BRT, followed by ITT and CK, all of which were significantly lower than that in HCT (χ^2^ = 57.075, *p<* 0.01; **Table 1**). Similarly, BRT had the lowest RSA, followed by ITT, whereas the RSA was significant higher in the HCT and control groups (χ^2^ = 46.99, *p<* 0.01).

**Table 1:** Comparable traits of root systems and three-level branches among the treatments.

Treatmenttype	Root length (mean, cm)	Root surface area (mean, cm^2^)	1^st^-level branch length (mean, cm)	2^nd^-level branch length (mean, cm)	3^rd^-level branch length (mean, cm)
χ^2^	57.075	46.99	16.412	30.912	9.506
*p-value*	0.000	0.000	0.001	0.000	0.023
CK	38.39 [12.16;90.39]^a^	12.74 [3.37;29.56]^a^	93.36 [77.27;120.17]^a^	51.52 [41.05;72.41]^a^	27.48 [16.19;37.05]^a^
BRT	29.34 [10.65;75.93]^b^	9.23 [2.52;22.18]^b^	80.60 [58.05;104.19]^b^	35.10 [24.20;52.25]^b^	22.67 [14.93;30.30]^ab^
ITT	31.94 [11.44;74.77]^b^	11.64 [2.21;25.89]^b^	82.76 [65.25;98.77]^b^	36.38 [26.14;47.30]^b^	19.21 [14.50;26.40]^b^
HCT	58.97 [18.85;109.60]^c^	15.55 [5.83;32.14]^ac^	59.78 [0.00;112.35]^b^	49.43 [32.60;72.76]^ab^	19.62 [12.50;33.85]^b^

Data are presented as median [25%;75% quartile] using Kruskal-Wallis test; the lowercase letters indicate significant differences among the treatments, *p*< 0.05; Treatment: CK~control, BRT~biased root, ITT~inclined trunk, HCT~half crown, the same below.

The lengths of the three levels of branches showed some similar differences among the treatments (**Table 1**). The shortest first-level branches were observed in HCT, followed by BRT and ITT, all of which were significantly lower than that in the control group (χ^2^ = 16.412, *p*< 0.01). The shortest second-level branches were observed in BRT, followed by ITT, both of which were significantly lower than that in CK (χ^2^ = 30.912, *p*< 0.01). The third-level branches showed the shortest in ITT, followed by HCT, both of which were significantly lower than that in CK (χ^2^ = 9.506, *p*< 0.05).

**Figure 4:**
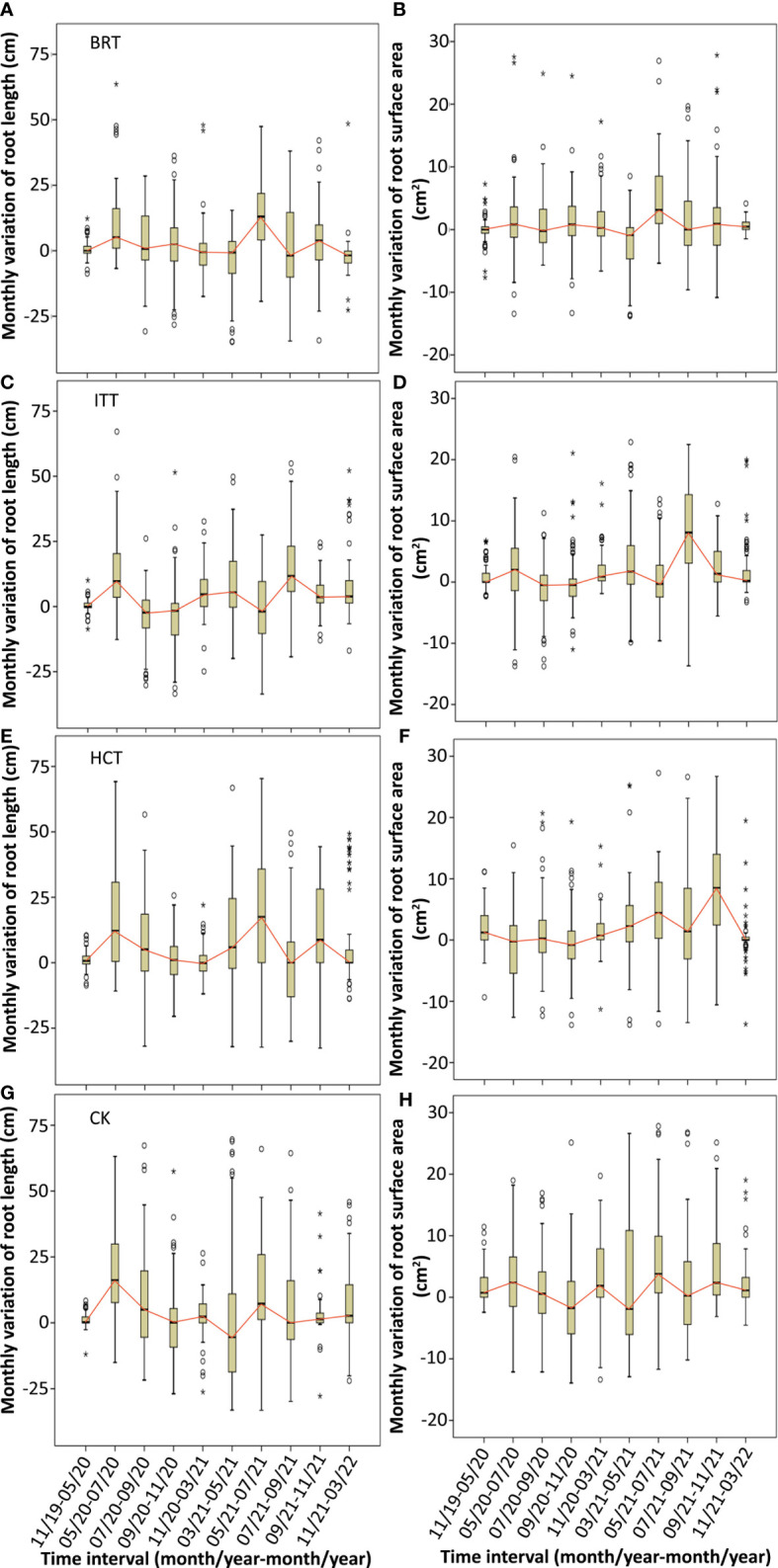
Monthly variation of root length **(A, C, E, G)** and root surface area **(B, D, F, H)** in the three treatments (BRT~**A, B**; ITT~**C, D**; HCT~**E, F**) and control **(G, H)**. Vertical boxes represent 50% of the observations (25th to 75th percentiles) and lines extending from each box are the upper and lower 25% of the distribution (90th and 10th percentiles). Within each box, the solid horizontal line is the median, the same below. The time interval was indicated as month/year-month/year. The years of 2019, 2020, 2021 and 2022 were abbreviated as 19, 20, 21 and 22 respectively, the same below.

Monthly variation in RL and RSA showed different growth dynamics among the treatments. The RL across treatments showed similar growth patterns, that is, one main peak during May–July (**Figures 4A, C, E, G**). BRT and ITT showed slightly lower growth curves than HCT and CK, whereas relative to CK, the HCT curve was lower in 2020 but higher in 2021. RSA showed a near bimodal curve in spring and autumn and a decrease in summer in the same year in all the treatments except HCT where the lower happened in spring 2020 (**Figures 4B, D, F, H**). BRT exhibited the lowest growth curve in 2021, whereas HCT first showed a lower growth curve, relative to CK, followed by a higher growth curve.

### 3.2 Response of biomass distribution of the crown to the asymmetric root system

**Figure 5:**
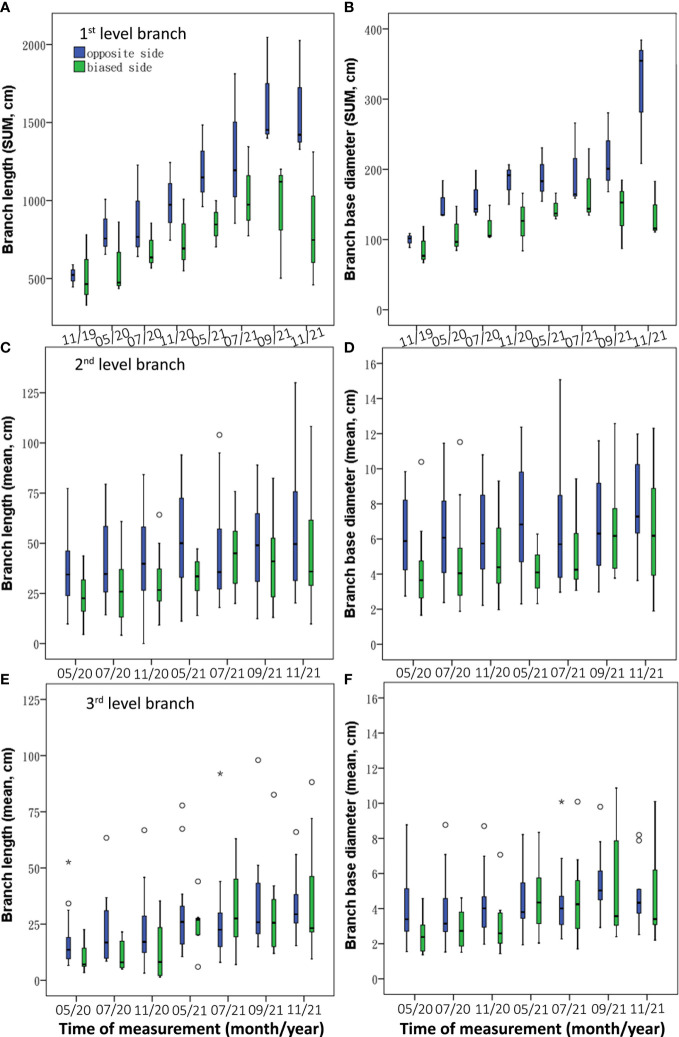
Temporal dynamics of branch variables [length **(A, C, E)** and base diameter **(B, D, F)**] between *0* and *1* sides under BRT treatment.

Asymmetric root treatment resulted in different patterns of branch growth between the *0* and *1* sides. The branch length was larger on the *0* side than on the *1* side, of which the first level and second level branches showed significantly longer on the *0* side (*Z* = –2.071 and –4.086 respectively, *p*< 0.05; **Table 2**). Furthermore, the sum of the first-level branches on the *0* side were longer during the experiment period (**Figure 5A**); the second-level branches were significantly longer on the *0* side during most of the experiment period except July 2021(**Figure 5C**); and the third-level branches were longer on the *0* side except that in May–July 2021(**Figure 5E**). The base diameters of three-level branches showed significantly higher on the *0* side (*Z* = –2.590, –4.508, and –2.351 respectively, *p*< 0.05). Furthermore, the three levels of branch kept the higher base diameter trend on the *0* side during the whole experimental period (**Figures 5B, D, F**).

**Table 2:** Directional comparisons of root and branch variables in response to the asymmetric treatments.

Directional division	Root length (mean, cm)	Root surface area (mean, cm^2^)	1^st^-level branch length (SUM, cm)	2^nd^-level branch length (mean, cm)	3^rd^-level branch length (mean, cm)
BRT					
*t/Z-value*			3.077^① (1)^	-4.086^② (2)^	-1.775^② (2)^
*p-value*			0.004	0.000	0.076
*0*			1112.6 (452.41)^a^	38.70 [27.13;59.85]^a^	20.70 [13.10;31.45]
*1*			776.56 (285.59)^b^	30.05 [21.20;43.13]^b^	20.00 [6.75;29.00]
ITT					
*t/Z-value*	-2.164^② (2)^	-1.142^② (2)^	2.049^① (1)^	3.044^① (1)^	0.967^① (1)^
*p-value*	0.030	0.251	0.157	0.003	0.337
*0*	34.57 [14.83;78.81]^a^	11.76 [3.24;25.01]	686.00 (292.71)^#^	44.95 (16.80)^#a^	22.43 (10.26)^#^
*1*	28.91 [9.22;70.37]^b^	11.29 [1.55;27.44]	589.33 (271.89)^#^	33.06 (15.61)^#b^	19.79 (11.52)^#^
HCT					
*Z-value*	-0.383^②^	-0.395^②^			
*p-value*	0.701	0.693			
*0*	58.70 [19.31;113.20]	16.25 [5.62;32.60]			
*1*	58.97 [18.21;107.81]	14.54 [6.08;30.97]			

Test method: ^①^T test, expressed as mean (standard deviation), ^②^Mann-Whitney U test, expressed as median [25%;75% quartile]; ^(1)^t value, ^(2)^Z value; the lowercase letters indicate the significant differences between directions; ^#^ the two-sided data took the reference line from the trunk; *0*~opoosite side, *1*~biased side.

**Figure 6:**
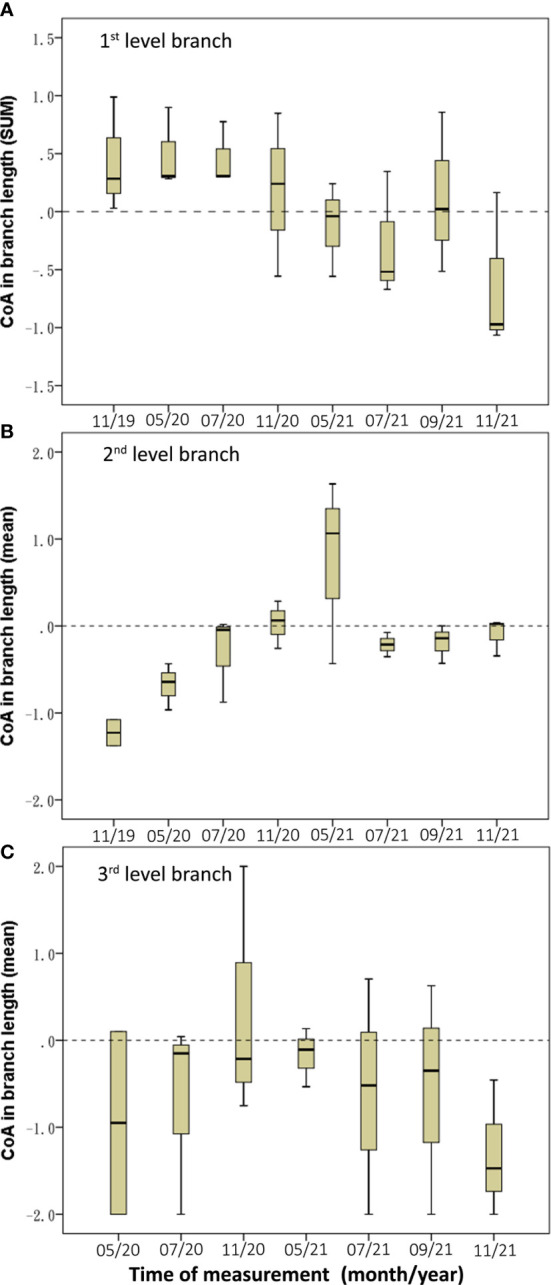
Temporal dynamics of length CoA of the three-level branches in BRT. **(A)** The 1st level branch, **(B)** the 2nd level branch, **(C)** the 3rd level branch.

The length CoAs of 2^nd^ and 3^rd^ level branches were negative under the asymmetric root treatment (Med = –1.05 and –1.28 respectively; **Table 3**). The CoAs of summed length of first-level branches were temporally transformed from positive values in 2020 to negative values in 2021(**Figure 6A**); the second-level branches had negative CoAs during the experiment period, except between November 2020 and March 2021 (**Figure 6B**); and the length CoAs of third-level branches were negative during the experiment period (**Figure 6C**).

**Table 3:** CoAs of root and branch traits between the treatments.

Treatmenttype	CoA in root length(mean)	CoA in root surface area (mean)	CoA in 1^st^-level branch length (SUM)	CoA in 2^nd^-level branch length (mean)	CoA in 3^rd^-level branch length (mean)
*t/Z-value*	-1.389^②(2)^	-1.194^①(1)^	-2.361	-1.915	-0.800
*p-value*	0.165	0.236	0.018	0.055	0.424
BRT			0.24 [-0.52;0.34]^a^	-1.05 [-1.54;-0.22]	-1.28 [-1.91;0.13]
ITT	-0.23 [-0.48;0.18]	-0.18(0.62)	-0.42 [-0.81;0.06]^#b^	-0.73 [-1.49;-0.18]^#^	-0.55 [-1.24;0.52]^#^
HCT	0.06 [-0.25;0.22]	-0.03(0.42)			
R_HCR_			-0.388*		

Test method: ^①^T test, expressed as mean (standard deviation), ^②^Mann-Whitney U test, expressed as median [25%;75% quartile]; ^(1)^t value, ^(2)^Z value, ^(4)^; the lowercase letters indicate the significant differences between the treatments, *p*< 0.05; CoA~coefficient of asymmetry; ^#^ the two-sided data took the reference line from the trunk; R_HCR_~Pearson correlation coefficient in HCT, *p < 0.05.

### 3.3 Response of biomass distribution of the root system to the inclined trunk and half-pruned crown

**Figure 7:**
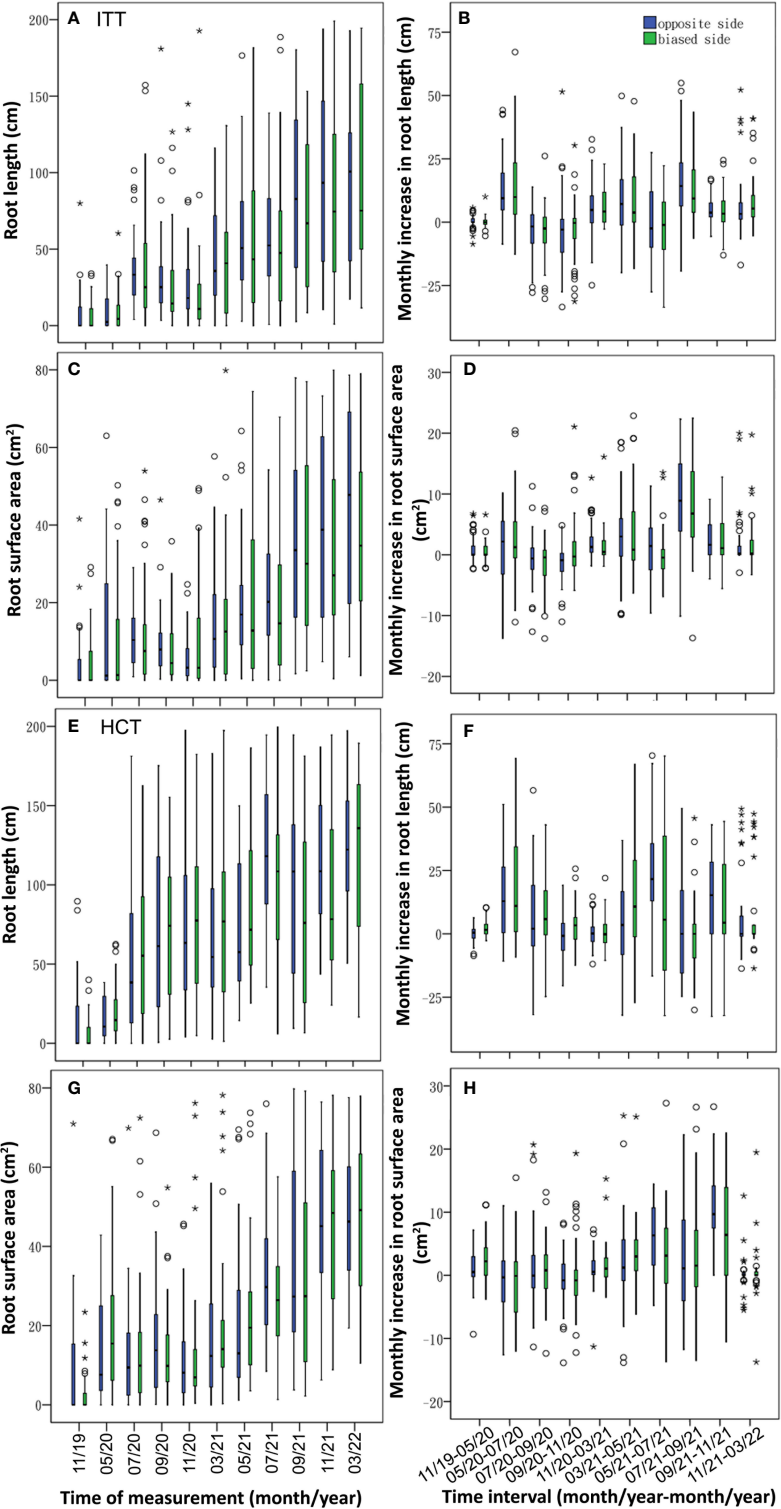
Temporal dynamics of root variables and their increment between *0* and *1* in ITT **(A–D)** and HCT **(E–H)**.

Both ITT and HCT induced asymmetric root growth, with a slight tendency for greater RL and RSA on the *0* side than on the *1* side, of which the RL was significantly larger on the *0* side in ITT (χ^2^ = –2.164, *p<* 0.05; **Table 2**). In ITT, the temporal dynamics showed longer roots on the *0* side starting in July 2020 except that in March 2021 (**Figure 7A**), and the monthly increase in root length growth was greater on the *0* side during three periods of July–September 2020, November 2020–May 2021, and July–November 2021 (**Figure 7B**). Greater RSA was observed on the *0* side from July to November 2020 and May 2021 till the end of the experimental period (**Figure 7C**), and the monthly increases of RSA on the *0* side were higher in May–July 2020 and November 2020–November 2021, wherein a two-sided significant difference was observed during May–September 2021 (*p<* 0.05; **Figure 7D**).

**Figure 8:**
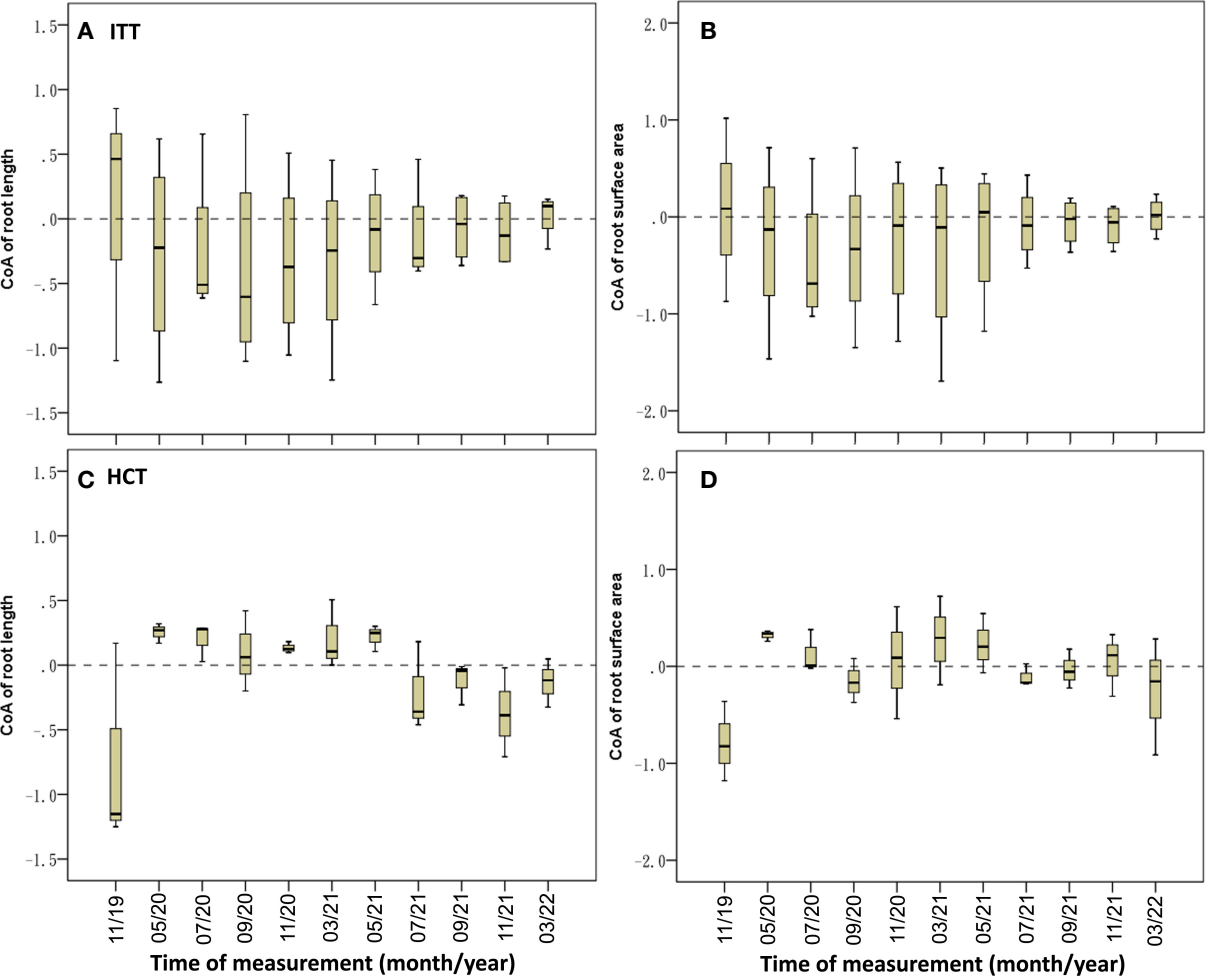
Temporal dynamics of CoA of root variables in ITT **(A, B)** and HCT **(C, D)**.

The median CoA_RL_ was –0.23, which remained negative during the experimental period except at the first and last sample collection, and the median CoA_RSA_ was –0.18, which remained negative except at the first and seventh sampling (**Figures 8A; B**; **Table 3**).

In HCT, RL and RSA did not show significant differences between *0* and/*1* sides. The RL was greater on the *0* side from May to November 2021 (**Figure 7E**), whereas the monthly increase in RL was observed higher on the *0* side in May–July 2020, November 2020–March 2021, May–July 2021, and September–November 2021 (**Figure 7F**). The RSA was larger on the *0* side during September 2020–March 2021, and July–September 2021 (**Figure 7G**), and the monthly increases in RSA were higher on the *0* side in May–July and September–November 2021 (**Figure 7H**).

The CoA_RL_ and CoA_RSA_ were negative in November 2019, and then transformed from positive to negative values in June 2021 (**Figures 8C, D**). The CoAs showed significant correlations between RL (Exp.) and the second-level branch (*R* = –0.388, *p<* 0.05; **Table 3**).

It is noted that three levels of branch showed longer on the *0* side than on the *1* side if the two-sided data took the reference line from the trunk in ITT (**Table 2**). The second-level and third-level branches were significantly longer on the *0* side than on the *1* side (*F* = 20.115, *p<* 0.01; F = 6.272, *p<* 0.05, respectively; **Table 2**), showing syntropic asymmetry of the root distribution. Despite this, most of crown body was distributed on the *1* side when the reference line was from the trunk base. The negative values of CoAs in RL and RSA showed an inverse relationship with the above-ground asymmetry.

### 3.4 Physiological responses to the asymmetric treatments

The leaf SOD content among the treatment groups was in the order: ITT > BRT > CK, but the differences were not statistically significant. ITT, where the stress of trunk bending weakened apical dominance, showed minimum *P_n_* and WUE and maximum content of soluble sugar, starch, and NSC. Furthermore, in ITT slightly higher SOD content was on the *0* side where there were significantly higher *P_n_*, WUE, and chlorophyll content (*Z* = 9.523, 23.517 and –2.656, respectively, *p<* 0.01; **Table 4**).

**Table 4:** Physiological variables among the treatments and their orientation traits.

Treatment	*P_n_* (μmol·m^-2^·s^-1^)	*T_r_* (mmol·m^-2^·s^-1^)	WUE (μmol·mmol^-1^)	Chl (mg·g^-1^)	TN (mg·g^-1^)	SS (mg·g^-1^)	Starch (mg·g^-1^)	NSC (mg·g^-1^)	SOD (mg·g^-1^)
*F/*χ^2^ *-value*	61.186^② (2)^	169.172^② (2)^	33.139^② (2)^	214.461^② (2)^	2.779^① (1)^	0.042^① (1)^	12.041^⑤ (1)^	14.457^⑤ (1)^	2.068^⑤ (1)^
*p-value*	0.000	0.000	0.000	0.000	0.171	0.848	0.000	0.000	0.222
CK	5.33 [3.69;6.53]^b^	2.20 [1.86;2.90]^c^	2.46 [1.27;2.81]^b^	32.30 [30.70;34.30]^c^	10.44 (1.37)^b^	44.49 (7.59)^b^	26.99 (4.93)^b^	71.48 (12.23)^b^	364.27 (136.65)
BRT	7.29 [5.39;9.77]^a^	4.64 [3.49;5.21]^a^	1.86 [1.18;2.27]^a^	39.60 [37.30;41.55]^a^	13.34 (1.50)^a^	32.10 (3.83)^a^	21.39 (2.39)^a^	53.49 (5.75)^a^	431.47 (79.85)
*Z/t -value*	2.655^① (4)^	-1.611^③ (3)^	-0.054^③ (3)^	-2.873^③ (3)^	-0.653^④ (4)^	0.029^④ (4)^	-0.536^③ (3)^	-0.197^③ (3)^	2.041^③ (3)^
*p-value*	0.109	0.107	0.957	0.004	0.549	0.979	0.621	0.854	0.115
*0*	6.54 (2.72)	3.66 [3.08;5.29]	2.20 [0.99;2.38]	37.90 [34.90;41.20]^a^	12.91 (1.86)	32.15 (4.33)	20.82 (2.47)	52.97 (5.84)	483.53 (54.30)
*1*	7.78 (3.05)	4.73 [4.10;5.17]	1.84 [1.32;2.10]	40.20 [39.10;41.75]^b^	13.76 (1.28)	32.05 (4.24)	21.95 (2.69)	54.00 (6.92)	379.41 (69.72)
ITT	4.96 [2.36;6.66]^b^	2.47 [2.08;3.08] ^b^	1.82 [1.25;2.18]^a^	35.15 [32.50;38.88]^b^	10.84 (1.99)^b^	48.06 (3.91)^b^	30.56 (1.41)^b^	78.62 (5.12)^b^	482.53 (74.68)
*Z/t -value*	9.523^③ (3)^	0.155^③ (3)^	23.517^③ (3)^	-2.656^③ (3)^	1.667^④ (4)^	0.205^④ (4)^	0.979^③ (3)^	0.406^③ (3)^	0.047^③ (3)^
*p-value*	0.002	0.693	0.000	0.008	0.171	0.848	0.388	0.710	0.965
*0*	5.56 [4.46;7.64]^a^	2.58 [2.22;3.26]	2.12 [2.03;2.46]^a^	37.00 [33.75;40.58]^a^	12.00 (1.94)	48.43 (3.24)	31.12 (1.18)	79.55 (4.18)	484.13 (104.61)
*1*	3.01 [1.92;6.55]^b^	2.86 [2.19;3.26]	1.48 [0.79;1.82]^b^	33.95 [32.40;36.78]^b^	9.68 (1.44)	47.70 (5.22)	29.99 (1.61)	77.69 (6.74)	480.92 (54.71)

Test method: ^①^one-way ANOVA test, expressed as mean (standard deviation), ^②^Kruskal-Wallis test, expressed as median [25%;75% quartile], ^③^Mann-Whitney U test, expressed as median [25%;75% quartile], ^④^T test, expressed as mean (standard deviation), ^⑤^Welch test, expressed as mean (standard deviation); ^(1)^F value, ^(2)^χ^2^ value, ^(3)^Z value, ^(4)^t value; the lowercase letters indicate the significant differences among the treatments or directions, *p*< 0.05; P_n_~net photosynthesis rate; T_r_~transpiration rate; WUE~water use efficiency; Chl~chlorophyll; TN~ total nitrogen; SS~soluble sugar; NSC~non-structural carbohydrates; SOD~superoxide dismutase; *0*~opoosite side, *1*~biased side.

BRT, which induced root system stress because of one-sided rooting constraint, had the highest values of leaf *P_n_*, *T_r_*, and chlorophyll and total nitrogen (TN) content (**Table 4**). However, BRT had the lowest content of soluble sugar, starch, and NSC among the treatments (**Table 4**), which may be the result of more photosynthates being preferentially used for morphogenesis (Du et al., 2020) to promote longer first- and third-level branches than those in ITT and HCT (**Table 1**). Slightly higher SOD on the *0* side showed moderate stress from the side of root restriction. In contrast, *P_n_* and *T_r_* were slightly higher on the *1* side. Furthermore, significantly higher value of chlorophyll content was observed on the *1* side (*Z* = –2.873, *p<* 0.05), which may be consistent with the nutrient allocation strategy.

Finally, the maximum WUE and minimum *T_r_*, chlorophyll content, and leaf TN values were observed in the control group, thereby indicating that this group had the highest nutrient conversion efficiency.

## 4 Discussion

### 4.1 Asymmetric treatments and asymmetric responses of biomass distribution

Tree root–shoot interactions have rich connotations in physics and physiology. The root/shoot ratio is used as an indicator to quantify the below- and above-ground interactions (Watson, 1991). Furthermore, the distribution of root biomass is related spatially to that of shoot morphology (Stokes et al., 1995). The root-shoot relationship should not only be reflected in the total biomass of root versus shoot but also in the structural distribution of biomass. Based on root-shoot mechanical balance, tree root system development respond spatially to the asymmetrical crown by shifting more root biomass to the opposite side of the asymmetry of crown (Stokes et al., 1995; Hardiman et al., 2017; Kolb et al., 2017). Furthermore, root–shoot structural interactions are relevant to water cycling and land-atmosphere gas exchange (Hardiman et al., 2017). Belowground biomass distribution is associated with nutrient uptake and retention and can in turn affect aboveground growth and structure (Parsons et al., 2016). Our study found that root growth was most suppressed in BRT, followed by ITT, and branch growth was most suppressed by HCT. Furthermore, we found greater branch length and diameter on the opposite side of the root-bias side in BRT, indicating opposite asymmetry between roots and shoots, as evident by the negative asymmetric coefficient. Similarly, we observed slightly greater RL and RSA values on the opposite side of the trunk-leaning side and half-crown side in both ITT and HCT, indicating opposite asymmetry between root and shoot traits, as evident by the negative asymmetric coefficients. It may be the result of the opposite asymmetric correlation between below- and above-ground biomass distributions of camphor tree, where asymmetric root systems can induce an asymmetric and opposite crown growth, and conversely, an asymmetric shoot can induce an asymmetric and opposite root growth. Similar results were observed by Stokes et al. (1995) who reported a spatial relationship between the root biomass distribution of *Picea sitchensis* and *Larix decidua* with that of shoot growth under wind loading. An asymmetric root system is induced on the side opposite to an asymmetric crown that is subject to wind, and following the biomechanical stability principle, is thus a key for tree anchorage. Dong et al. (2019) found that half-crown pruning of *Cunninghamia lanceolata* induced greater decreases in RL on the pruned side than on the opposite side of the crown, whereas half-root pruning led to bilateral difference in branch length that transformed from significant at 10 weeks after pruning to non-significant at 60 weeks. Our study not only supports the hypothesis that the asymmetry of root formation is related to aboveground architectures but also that the asymmetric root system can influence aboveground formations. Our study may be the first to provide novel insights into the converse nature of specific root–shoot interactions in tree architecture.

### 4.2 Comparison between stress in below- and above-ground organs

The root–shoot interactions in tree architecture should be supported by physical and physiological variables. Optimal partitioning theory has become the basis for predicting plant growth responses to multiple external stresses (Gedroc et al., 1996; Kobe et al., 2010; Yan et al., 2016). Per this theory, plants need to balance the allocation of nutrients across organs to achieve the most efficient and preferable allocation under stresses (Bever, 2015). More nutrients will be translocated into roots when the below-ground organs are under stress, whereas more nutrients will be allocated to the shoot system when the above-ground organs are under stress (Poorter et al., 2012). For example, plants will allocate more nitrogen to leaves to compensate for the low photosynthetic rate in arid conditions (Yan et al., 2016), and distribute a relatively higher proportion of biomass to roots when mineral elements are scarce (Hermans et al., 2006). Sheltered plants allocate biomass mostly to shoots, whereas the plants without shelter invest more resources into the root system (Mariotti et al., 2015). Trunk leaning mitigates growth vigor and reduces leaf nitrogen content and carbohydrate output so as to increase the content of soluble sugar (Wang et al., 2013). Our study found maximum NSC_leaf_ values in ITT and minimum NSC_leaf_ values in BRT, likely owing to stress from above and below the ground, respectively, which is consistent with the optimal partitioning theory. The differential nutrition allocation demonstrates allometric relation and the idea that plants can adjust the relative nutrient distributions in below- and above-ground organs. Furthermore, the differences can be exhibited as asymmetry of one organ when only one side of a tree experiences stress.

One-sided rooting constraint resulted in the asymmetrical distribution of root systems in BRT, which induced the asymmetrical growth transformation of the crown from longer first-level branches on the root-bias side during the initial months to that on the opposite side during the later months. The earlier stage indicates equivalent asymmetry of roots and shoots, in alignment with the nutrient allocation strategy, and the later stage indicates opposite asymmetry of roots and shoots, in accordance with the biomechanical stability principle. The lower NSC content on the opposite side may imply that more photosynthates were preferentially used for tree morphogenesis to generate more biomass.

In ITT, the leaning trunk changed the crown mass center, resulting in tree asymmetry that could decrease tree stability (Strigul, 2012). However, the trees can reorient owing to gravitropism, as evidenced in the apices of maritime pine saplings inclined at >30° (Herrera et al., 2010). Under the effect of negative gravitropism, the lateral branches grow longer and more outwardly from the leaning side, tending toward the erect axis. We observed that root asymmetry is proportional to crown asymmetry, which is consistent with both the nutrient allocation strategy and biomechanical stability principle. Higher values of SOD, *P_n_*, WUE, and NSC content on the opposite side indicate that stress can induce higher production of photosynthates on that side for morphogenesis.

In HCT, the induced root asymmetry appeared to transform from longer roots on the half-crown side during the earlier months to longer roots on the opposite side during the later months. This trend was similar to that observed in BRT, in the sense that the equivalent asymmetry of roots and shoots occurred during the earlier growth stage, consistent with the nutrient allocation strategy, and the opposite asymmetry of roots and shoots occurred during the later growth stage, as per the biomechanical stability principle.

### 4.3 Root–shoot interactive correlations of biomass distribution with potential use in root design

Compared to natural forests, urban trees frequently encounter harsher ecological environments (Kontogianni et al., 2011), due largely to the presence of the impervious surfaces of buildings, roads, driveways, streets, and parking lots. The below- and above-ground growth space of many street trees are often narrow, resulting in tree weakness and even mortality (Limoges and Apparicio, 2018; Yan et al., 2019). Asymmetric tree architecture can be found near buildings, driveways, and river banks in urban areas, which also detracts from tree stability (Rahardjo et al., 2009; Volder et al., 2009; Kontogianni et al., 2011; Bobrowski et al., 2017). Definite asymmetric correlation between the biomass distribution of the root system and shoots is the key to understanding the spatial interactions of below- and above-ground biomass. In this study, we found that camphor tree demonstrates opposite root–shoot asymmetries under various stresses, which is essential when designing the optimum space for root development in anticipation of likely asymmetrical above-ground tree architecture. For example, a camphor tree is expected to have larger canopies oriented toward street for shading; thus, its potential root system can be designed in a larger space away from the street to achieve more equalized growth patterns. Therefore, we can predict crown morphology of an aged tree from the sapling root habitat. Scientific root design will benefit silvicultural management of urban forests and promote healthier ecological environments in urban areas.

## 5 Conclusion

Here, we conducted a controlled cultivation experiment on camphor saplings under asymmetric treatments of roots and shoots. BRT included asymmetry of crown growth, whereas ITT and HCT included asymmetry of the root system. Leaf nutrient contents (e.g., TN, chlorophyll, soluble sugar, starch, and NSC) and physiological variables (e.g., *P_n_*, *T_r_*, WUE, and SOD) supported the characteristics of biomass distribution; the interactive correlation between below- and above-ground organs observed here can be used in root design when planning urban forests.

Nevertheless, data were incomplete because of the interruptions in the study period due to COVID-19. Additionally, non-significant results were obtained when comparing the RL and RSA on the *0* side with those on the *1* side in ITT and HCT as well as when comparing first-level branches on the two sides in BRT. Further studies should be conducted over longer periods to verify the two-sided architectural evolution based on the development of fine roots. Similar experiments should be carried out on other tree species to generalize the root–shoot architectural relationship.

## Data availability statement

The raw data supporting the conclusions of this article will be made available by the authors, without undue reservation.

## Author contributions

HW, YH, and JQ designed the experiments. CG, DW, QX, LP, KX, YS, JG, and RJ collected data and performed the analysis. HW, YH, and JQ drafted the manuscript. All authors critically revised and approved the final version of this manuscript.

## Funding

This study was supported by the Shanghai Municipal Project of the Committee of Science and Technology (Grant No. 21DZ1202000, 21DZ1202003), and Shanghai Municipal Administration of Greening and Appearance Project (Grant No.G212409).

## Acknowledgments

We would like to thank Drs. Chai-Shian Kua, Luke McCormack, Chuck Cannon, and Gary Watson from Morton Botanical Garden, USA for their help in experiment design. We would give thanks to Profs. Jun Yang from Tsinghua University, Jiakuan Chen from Fudan University, Zhengquan Wang from Northeast Forestry University, and Shuiliang Guo from Shanghai Normal University for their professional consultations. We also thank the reviewers for their valuable suggestions.

## Conflict of interest

The authors declare that the research was conducted in the absence of any commercial or financial relationships that could be construed as a potential conflict of interest.

## Publisher’s note

All claims expressed in this article are solely those of the authors and do not necessarily represent those of their affiliated organizations, or those of the publisher, the editors and the reviewers. Any product that may be evaluated in this article, or claim that may be made by its manufacturer, is not guaranteed or endorsed by the publisher.

## Abbreviations

CoA, coefficient of asymmetry; BRT, biased-root treatment; HCT, half-crown treatment; ITT, inclined-trunk treatment; NSC, non-structural carbohydrates; *P_n_*, net photosynthesis rate; RL, root length; RSA, root surface area; SOD, superoxide dismutase; *T_r_*, transpiration rate; WUE, water use efficiency.
