# Supplementary material for: Interactive responses of root and shoot of camphor tree (Cinnamomum camphora L.) to asymmetric disturbance treatments
Source: Front Plant Sci. 2022 Nov 29;13:993319. doi: 10.3389/fpls.2022.993319 (PMC9744769; doi:10.3389/fpls.2022.993319)
Supplement: Supplementary file 1 [file Image_1.pdf]

Appendix 1 Photos of experiment field

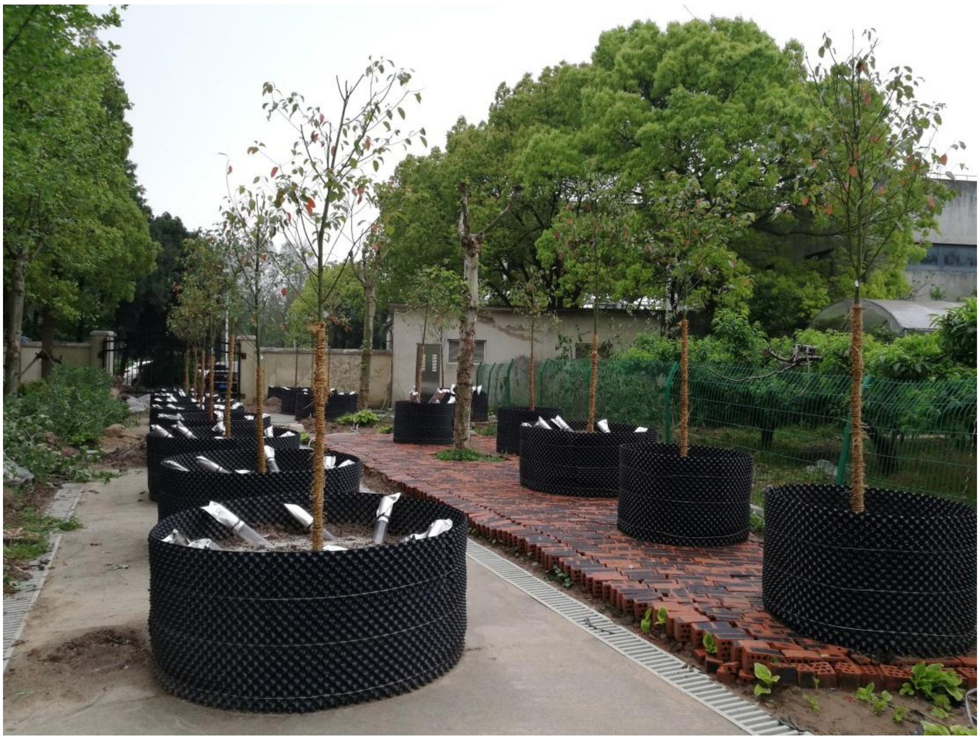

Photo 1: Experiment field, 04-2019

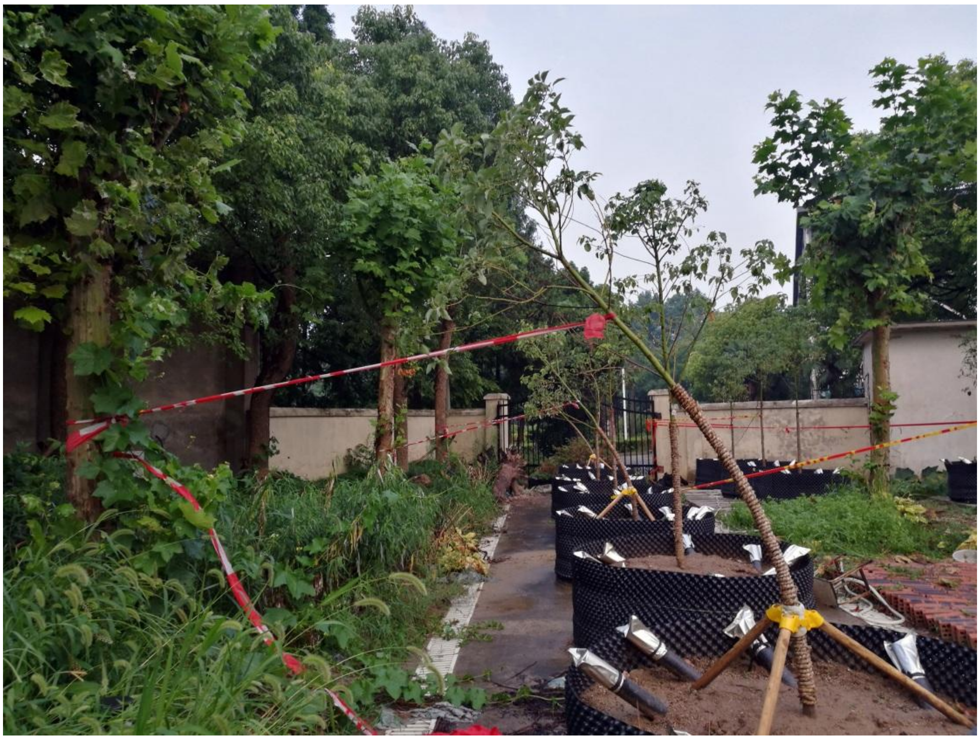

Photo 2: Inclined-trunk treatment, 07-2019

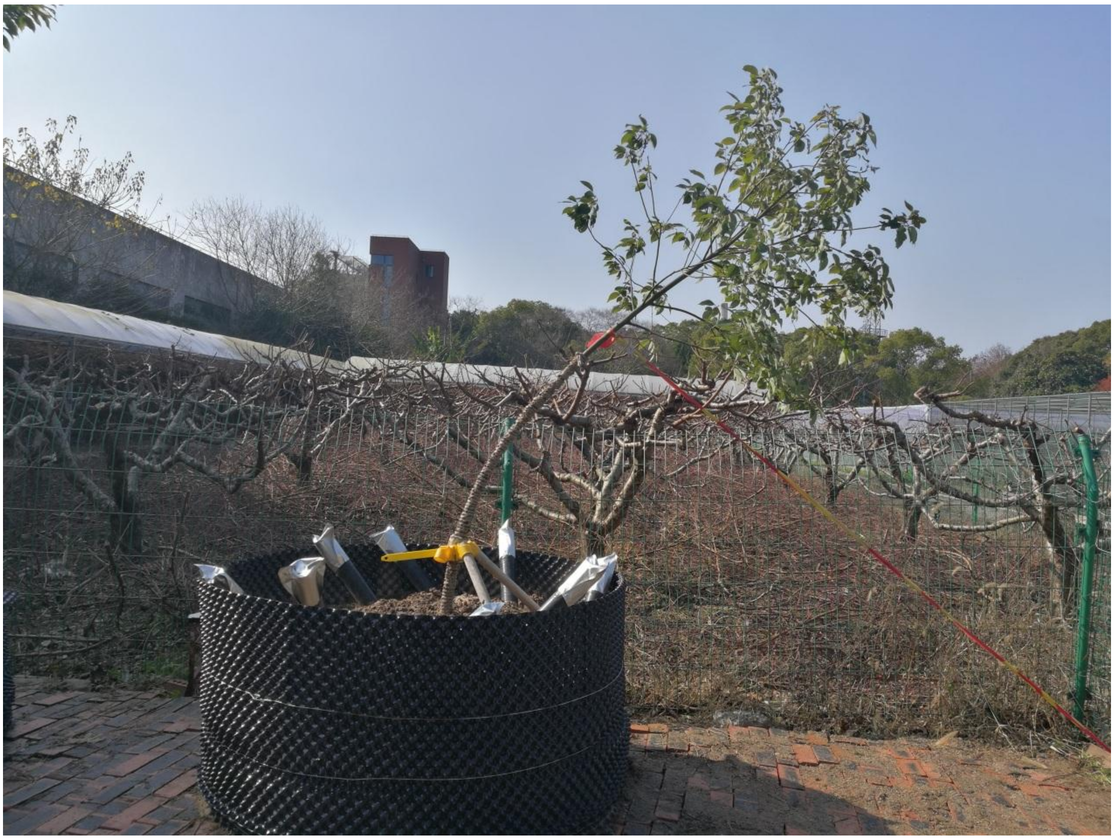

Photo 3: The tree added as an inclined-trunk treatment, 01-2020

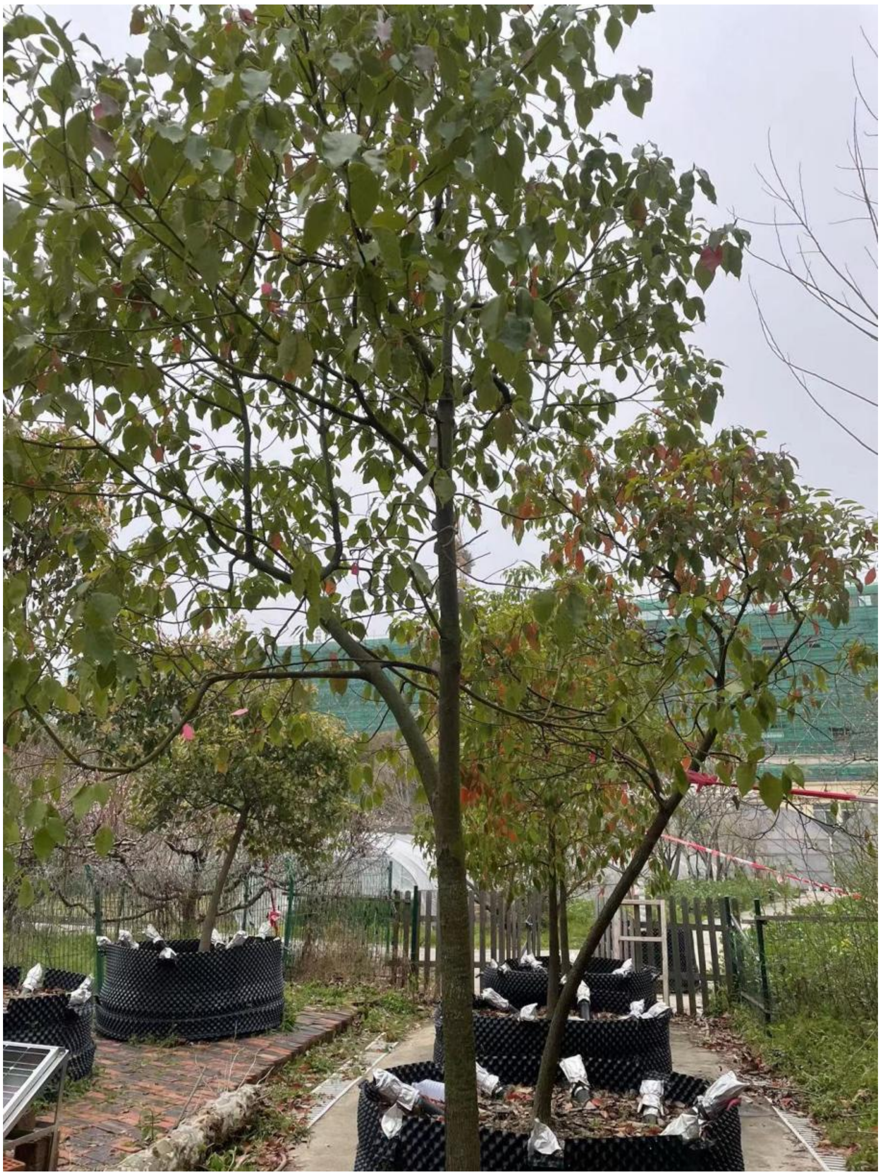

Photo 4: Half crown treatment

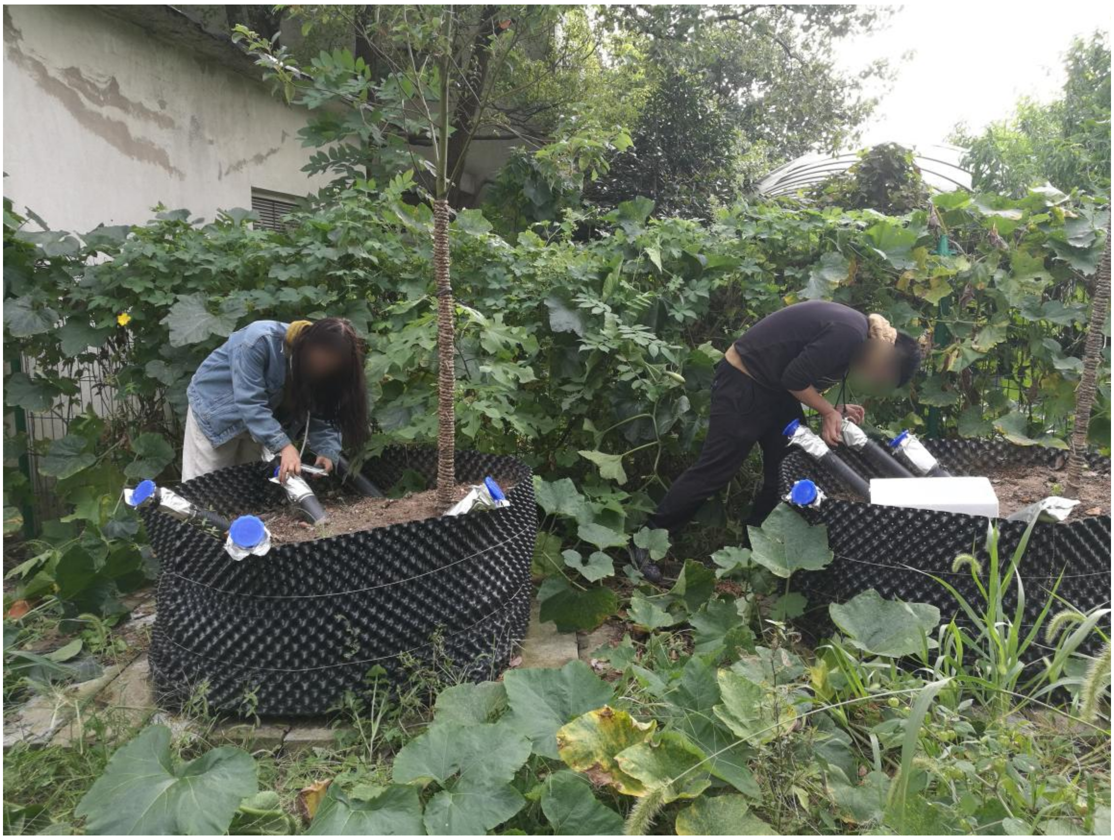

Photo 5: Biased root treatment

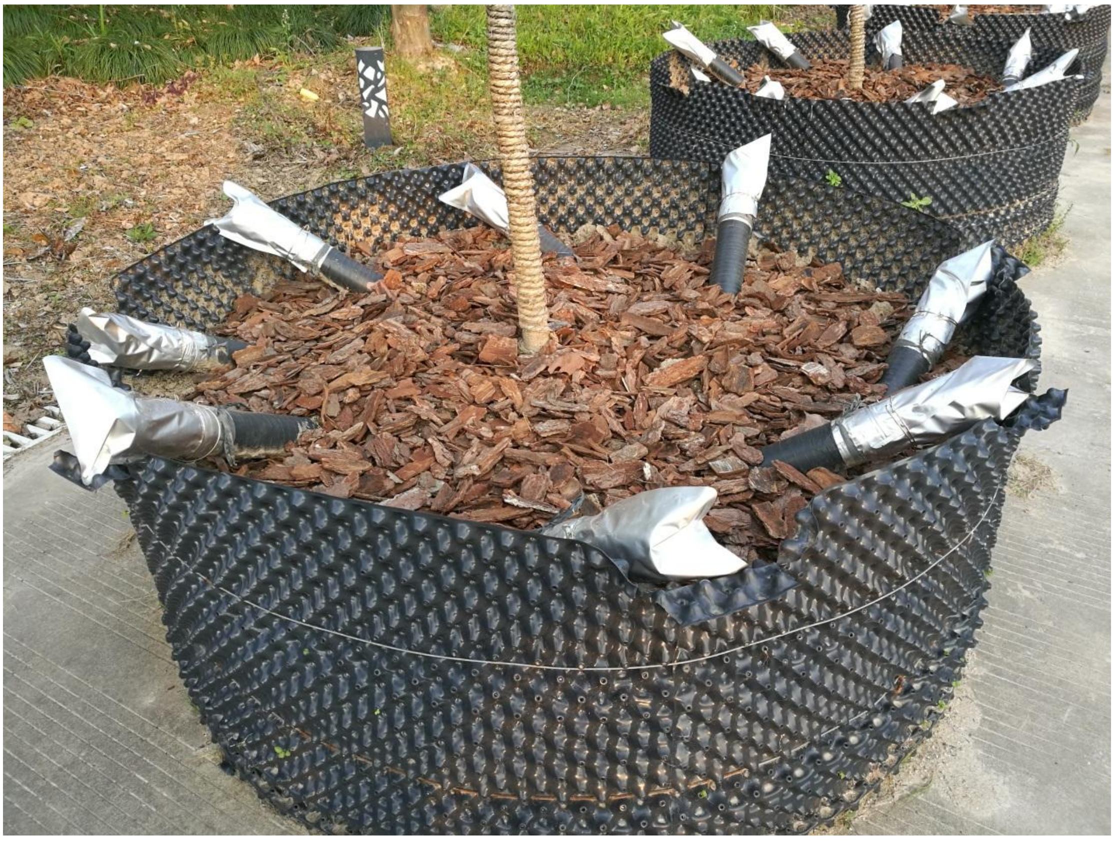

Photo 6: Layout of rhizotron tubes
